# Biochemical characterization of an enantioselective esterase from *Brevundimonas* sp. LY-2

**DOI:** 10.1186/s12934-017-0727-4

**Published:** 2017-06-19

**Authors:** Jing Zhang, Mengjun Zhao, Die Yu, Jingang Yin, Hao Zhang, Xing Huang

**Affiliations:** 1 Key Laboratory of Agricultural Environmental Microbiology, Ministry of Agriculture, College of Life Sciences, Nanjing Agricultural University, Nanjing, 210095 China; 2 Institute of Agricultural Resources and Environment, Jiangsu Academy of Agricultural Sciences, Nanjing, 210014 China; 3 Laboratory of Biocatalysis and Synthetic Biotechnology, State Key Laboratory of Bioreactor Engineering, East China University of Science and Technology, Shanghai, 200237 China

**Keywords:** Esterase, Lactofen, *Brevundimonas* sp., Enantioselective degradation

## Abstract

**Background:**

Lactofen, a member of the diphenylether herbicides, has high activity and is commonly used to control broadleaf weeds. As a post-emergent herbicide, it is directly released to the environment, and easily caused the pollution. This herbicide is degraded in soil mainly by microbial activity, but the functional enzyme involved in the biodegradation of lactofen is still not clear now.

**Results:**

A novel esterase gene *lacH*, involved in the degradation of lactofen, was cloned from the strain *Brevundimonas* sp. LY-2. The gene contained an open reading frame of 921 bp, and a putative signal peptide at the N-terminal was identified with the most likely cleavage site between Ala 28 and Ala 29. The encoded protein, LacH, could catalyze the hydrolysis of lactofen to form acifluorfen. Phylogenetic analysis showed that LacH belong to family V of bacterial lipolytic enzymes. Biochemical characterization analysis showed that LacH was a neutral esterase with an optimal pH of 7.0 and an optimal temperature of 40 °C toward lactofen. Besides, the activity of LacH was strongly inhibited by Hg^2+^ and Zn^2+^. LacH preferred short chain *p*-nitrophenyl esters (C_2_–C_6_), exhibited maximum activity toward *p*-nitrophenyl acetate. Furthermore, the enantioselectivity of LacH during lactofen hydrolysis was also studied, and the results show that *R*-(−)-lactofen was degraded faster than *S*-(+)-lactofen, indicating the occurrence of enantioselectivity in the enzymatic reaction.

**Conclusions:**

Our studies characterized a novel esterase involved in the biodegradation of diphenylether herbicide lactofen. The esterase showed enantioselectivity during lactofen degradation, which revealed the occurrence of enzyme-mediated enantioselective degradation of chiral herbicides.

**Electronic supplementary material:**

The online version of this article (doi:10.1186/s12934-017-0727-4) contains supplementary material, which is available to authorized users.

## Background

Many pesticides in current use contain chiral structures, which consist of enantiomers [1]. Enantiomers of a chiral compound exhibit identical physical and chemical properties [2]. However, their toxicities, biological activities and environmental fates vary because biological processes usually show high enantioselectivity [3, 4]. Enantioselective analysis is required for a full understanding of the biological behavior of chiral compounds. Information on the enantioselective degradation dynamics of chiral pesticides will help us to evaluate the impacts of such pesticides to environment. Enantioselective degradation in soils has been observed for various chiral pesticides, such as mecoprop, dichlorprop, metalaxyl and malathion [5–7]. When one enantiomer is preferentially degraded, the enantiomeric ratio [ER, the ratio of the concentration of (+)-enantiomers to (−)-enantiomers] deviates from the original value [2, 8]. The occurrence of such selective degradation involves the mediation of bacteria, enzymes, or other biological entities.

Diphenyl ether herbicides are widely used to control broadleaf weeds in cereal crops, soybeans, peanuts, and potatoes [9]. This class of herbicides has been proved to inhibit protoporphyrinogen oxidase, thereby resulting in the accumulation of protoporphyrin and blockage of chlorophyll formation [10, 11]. Their frequent occurrence in natural waters and soils indicate that they may be important environmental contaminants [12–14]. Lactofen is an important member of the diphenyl ether family. It has one chiral center in the alkyl moiety and consists of two enantiomers. This herbicide is applied as a racemic mixture, although its herbicidal activity is almost entirely attributed to the *S*-enantiomer [15]. The enantioselective degradation of lactofen in soil and sediment has been studied by Diao et al. [15, 16]. The *S*-enantiomer was preferentially degraded either in soil or sediment, resulting in a relative enrichment of the *R*-enantiomer. However, to our knowledge, no studies have focused on the enantioselectivity of the degrading-enzyme involved in lactofen metabolism so far.

The lactofen-degrading strain *Brevundimonas* sp. LY-2 was isolated from enrichment cultures inoculated with lactofen-contaminated soil sample in our lab. This strain could degrade about 80% of 50 mg L^−1^ lactofen in 5 days of incubation in flasks. The metabolic behaviors of the herbicide in the media were described [17]. In this study, we have found that the degradation process of lactofen by LY-2 is enantioselective, with *R*-(−)-lactofen being degraded faster than the *S*-(+)-enantiomer, which implies that the enzyme involved in lactofen degradation probably has the enantioselectivity between the different enantiomers. In this study, a novel esterase gene *lacH* involved in lactofen degradation was cloned from this strain, and the biochemical properties of the purified enzyme LacH were determined. The enantioselectivity of LacH during lactofen hydrolysis was also investigated.

## Results

### Cloning and sequence analysis of the esterase gene

The total DNA was extracted from strain *Brevundimonas* sp. LY-2, and the genomic library was constructed. A positive clone that produced a transparent halo around the colony was screened from approximately 3000 transformants. The sequencing results show that the inserted fragment in the transformant was 3453 bp containing three complete ORFs. These ORFs were each subcloned into the pMD18-T vector and then transformed into competent *Escherichia coli* DH5α cells. One ORF, designated as *lacH*, was confirmed to be the target gene encoding the lactofen-hydrolyzing enzyme. This ORF consists of 921 bp that encode a protein with 306 amino acids. A putative signal peptide at the N-terminal was identified by the SignalP 4.1 server, with the most likely cleavage site situated between amino acids Ala 28 and Ala 29. Thus, the encoded protein, LacH, is a secretory protein. The BLAST results showed that LacH shared moderate identities with some characterized α/β-hydrolase fold proteins, and showed the highest identity (71%) with esterase sys410 (AFE88176), a thermostable pyrethroid-hydrolyzing enzyme isolated from uncultured bacterium through the metagenomic approach [18]. Among the other characterized proteins, LacH shared 48% identity with an α/β-hydrolase fold protein from *Caulobacter crescentus* CB15 (AAK24201) [19], 40% identity with a triacylglycerol lipase from *Moraxella* sp. TA144 (CAA37863) [20], and only 27% identity with a triacylglycerol lipase from *Psychrobacter immobilis* B10 (CAA47949) [21].

**Fig. 1:**
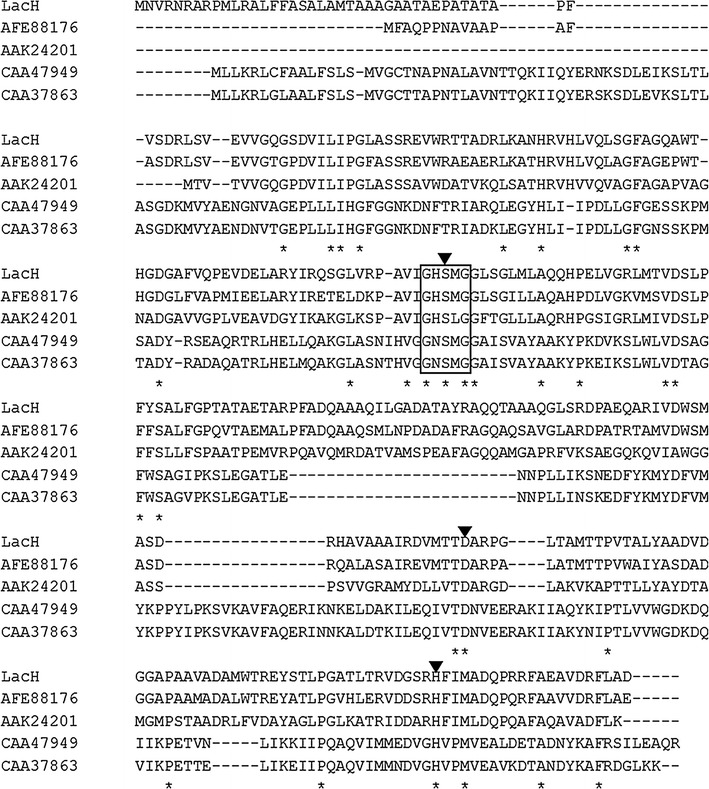
Multiple alignment of LacH with other related proteins. Except for LacH (this study), all other protein sequences were retrieved from GenBank (NCBI). AFE88176, esterase sys410 from uncultured bacterium; AAK24201, α/β-hydrolase fold family from *Caulobacter crescentus* CB15; CAA47949, triacylglycerol lipase from *Psychrobacter immobilis* B10; CAA37863, triacylglycerol lipase from *Moraxella* sp. TA144. The conserved pentapeptide of GXSXG is *boxed*. *Triangles* indicate the putative catalytic triad of serine, aspartate, and histidine residues. Identical amino acid residues are marked by *asterisk*

**Fig. 2:**
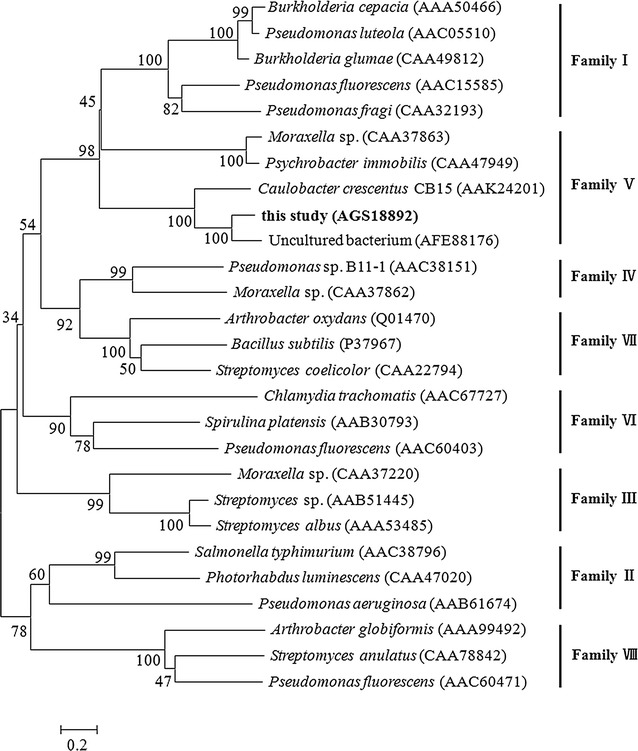
Phylogenetic analysis of LacH and related esterase/lipase proteins. Phylogenetic relationship of LacH and esterase/lipase proteins of eight different families was performed using the program MEGA 6.0. Except for LacH, all other protein sequences were retrieved from GenBank (NCBI). The *scale at the bottom* represents the number of substitution events

Multiple sequence alignment of LacH and other esterase/lipase proteins demonstrates that LacH contained a typical Ser-His-Asp/Glu (Ser128, Asp233 and His286) catalytic triad and also contained the conserved GXSXG motif (residues 126–130) (Fig. 1) [22, 23]. Bacterial esterases/lipases have been classified into eight different families based on their amino acid sequences and biochemical properties [24]. A phylogenetic tree was constructed to verify the evolutionary relationships between LacH and other known esterases/lipases. The phylogenetic tree showed that the LacH protein belonged to family V of lipolytic enzymes (Fig. 2).

### Expression and purification of the recombinant LacH

**Fig. 3:**
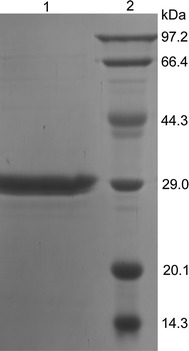
SDS-PAGE analysis of the purified LacH. *Lane 1* the purified LacH. *Lane 2* protein markers

The *lacH* gene, minus the signal peptide, was cloned into the expression vector pET-29a (+) to generate pET-LacH and expressed in *E. coli* BL21 (DE3) with a C-terminal His-tag. Recombinant LacH was purified from the crude extract using Ni-nitrilotriacetic acid affinity chromatography. The molecular mass of the denatured enzyme was approximately 30 kDa, as showed by SDS-PAGE (Fig. 3), which matched the predicted molecular mass (29,451 Da). Gel filtration indicated a molecular mass of 62 kDa. Comparison of this value with the calculated molecular mass suggested that this enzyme was a dimer.

### Characteristics of LacH

**Fig. 4:**
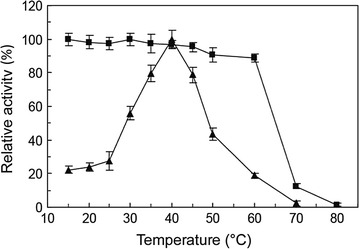
Effect of temperature on the activity (*triangles*) and stability (*boxes*) of LacH. The activity of the enzyme at different temperatures was measured in 50 mM sodium phosphate buffer (pH 7.0) using lactofen as the substrate. For determination of the thermostability, the enzyme was preincubated at various temperatures ranging from 15 to 80 °C for 30 min, and the residual activity was determined at pH 7.0 and 40 °C

**Fig. 5:**
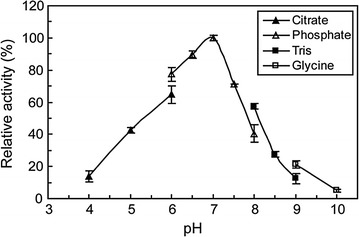
The effect of pH on the activity of LacH. The activity of the enzyme at different pH was measured at 40 °C

The effect of temperature on enzyme activity was examined in the range of 15–70 °C (Fig. 4). The maximum LacH activity was observed at 40 °C, and the minimal enzyme activity was observed at temperatures above 60 °C. Thermostability was determined by analyzing the residual enzyme activity after pre-incubation at temperatures ranging from 15 to 80 °C for 30 min (Fig. 4). The enzyme was fairly stable up to 60 °C, and retained approximately 88% of its activity at 60 °C for 30 min. Moreover, LacH retained only 12% of its activity at 70 °C, and was completely inactivated at 80 °C. These results showed that LacH was a potential thermostable esterase. The optimal pH of LacH was determined at 40 °C from pH 4.0 to pH 10.0 (Fig. 5). LacH showed the highest activity at neutral pH, with more than 80% of its maximal activity from pH 6.5 to pH 7.0.

**Fig. 6:**
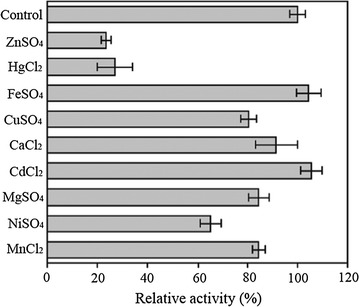
The effects of metal ions on the activity of LacH. Control reaction was carried out in the absence of metal ion

The effects of metal ions on enzyme activity were determined using various metal ions at 1 mM (Fig. 6). Activity of LacH was strongly inhibited by more than 70% with the presence of Hg^2+^ and Zn^2+^, whereas the presence of Ni^2+^ caused approximately 35% inhibition. Moreover, the addition of Mn^2+^, Mg^2+^, Ca^2+^, and Cu^2+^ showed only slight effects on the enzyme activity (10–20% inhibition).

### Substrate specificity and activity of LacH

The substrate specificity of LacH was determined using *p*-nitrophenyl esters with various acyl chain lengths (C_2_, C_4_, C_6_, C_8_, and C_12_) as substrates (Table 1). LacH showed the highest activity with *p*-nitrophenyl acetate (C_2_) (118 μmol min^−1^ mg^−1^) and the activity decreased when the aliphatic chain length of the substrate increased. The activity toward *p*-nitrophenyl caprylate (C_8_) in the assay was observed to be very low, and no significant esterase activity was observed for the *p*-nitrophenyl laurate (C_12_) under the same assay conditions. For C_2_, the *k*_cat_ and *K*_m_ values were 8.39 s^−1^ and 0.147 mM, respectively. With the increase in aliphatic chain length from 4 to 12 (C_4_–C_12_), *k*_cat_ decreased, whereas *K*_m_ increased.

**Table 1:** Substrate specificity and kinetic constants of LacH

Substrate	Specific activity (μmol min^−1^ mg^−1^)	*k*_cat_ (s^−1^)	*K*_m_ (mM)	*k*_cat_/*K*_m_ (s^−1^ mM^−1^)
Lactofen	2.97 ± 0.61	0.87 ± 0.09	0.067 ± 0.002	12.9
*p*-Nitrophenyl acetate	118 ± 5.3	8.39 ± 0.28	0.147 ± 0.008	50.1
*p*-Nitrophenyl butyrate	88.1 ± 3.2	7.48 ± 0.45	0.185 ± 0.009	40.4
*p*-Nitrophenyl caproate	15.8 ± 1.2	3.47 ± 0.13	0.208 ± 0.012	16.7
*p*-Nitrophenyl caprylate	4.90 ± 0.18	0.767 ± 0.06	0.335 ± 0.016	2.29
*p*-Nitrophenyl laurate	ND	ND	ND	ND

*ND* not detectable

Degradation efficiency by LacH with lactofen as the substrate was tested by high-performance liquid chromatography (HPLC) analysis (Additional file 1: Figure S1). The hydrolysis rate of lactofen was 59.3% under assay conditions of pH 7.0 and 40 °C for 15 min (Additional file 1: Table S1). The metabolite of lactofen hydrolysis by LacH was confirmed to be acifluorfen (Additional file 1: Figure S1).

### Enantioselectivity of LacH

**Fig. 7:**
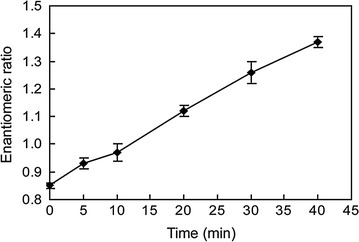
ER changes in the enzymatic reaction with lactofen as the substrate. The enantiomeric ratio [ER = *S*-(+)-lactofen/*R*-(−)-lactofen]

The enantioselectivity of LacH in the enzymatic reaction was investigated by evaluating the changes in enantiomer composition for lactofen. Enantiomers of lactofen were completely separated by HPLC with a chiral column (Additional file 1: Figure S2). ER [ER = *S*-(+)-lactofen/*R*-(−)-lactofen] was adopted as the standard descriptor [2]. At the initial point of the reaction, no substrate was hydrolyzed, and the initial ER was 0.85. When the enzymatic reaction proceeded, the ER values gradually increased from 0.85 to 1.37 (Fig. 7). This steady increase in ER in the enzymatic reaction suggested that the *R*-(−)-lactofen was preferentially degraded compared with the *S*-(+)-lactofen. The stereospecific enzymatic reaction implies that LacH involved in the conversion of lactofen could differentiate the *S*-(+)-lactofen from the *R*-(−)-lactofen.

## Discussion

Esterases (EC 3.1.1.1) and lipases (EC 3.1.1.3) are widely distributed in animals, plants and microorganisms. They have played an important role in the hydrolysis of a wide range of xenobiotics containing ester bonds. Esterases and lipases found in bacteria have been divided into eight different families based on their amino acid sequences and biochemical properties [24]. The esterase LacH that was cloned from strain LY-2 was responsible for converting lactofen to acifluorfen. Cheng et al. [25] studied the acute toxicity of lactofen and its metabolite acifluorfen in the aquatic algae *Scenedesmus obliquus*, and the results indicated that the toxicity of the acifluorfen was lower than that of the lactofen. Wang et al. [26] evaluated the toxic effects of the herbicide lactofen and acifluorfen to the aquatic plant *Lemna minor*, and they found *L. minor* growth was inhibited in the order of lactofen > acifluorfen. Both of these studies have found that acifluorfen had a lower toxicity to aquatic organisms than lactofen. To the best of our knowledge, LacH is the first identified esterase for lactofen hydrolysis in microorganisms. LacH belonged to the α/β-hydrolase fold protein family and contained both the typical Ser-His-Asp/Glu (Ser128, Asp233 and His286) catalytic triad and the conserved pentapeptide sequence GXSXG (residues 126–130) of α/β-hydrolase fold proteins [22, 23]. Amino acid sequence alignment and phylogenetic analysis suggested that LacH belonged to family V of lipolytic enzymes.

The substrate spectrum of LacH was found to be broad. It was able to hydrolyze various *p*-nitrophenyl esters of short-medium chain fatty acids (C_2_–C_8_). The catalytic efficiency value (*k*_cat_/*K*_m_) demonstrated that *p*-nitrophenyl acetate (C_2_) was the most efficient catalytic substrate. The specificity profile indicated that LacH should be classified as an esterase because maximal activity was observed on the short-chain fatty acid esters, while the activity of long-chain fatty acid esters was low. LacH could also hydrolyze other pesticides with similar carboxyl ester, such as fenoxaprop-*P*-ethyl, cyhalofop-butyl, fluazifop-*P*-butyl, quizalofop-*P*-ethyl and fluoroglycofen (Additional file 1: Table S1).

The degradation process of lactofen by *Brevundimonas* sp. LY-2 is enantioselective, with *R*-(−)-lactofen being degraded faster than the *S*-(+)-enantiomer (Additional file 1: Table S2), which implies that the enzyme involved in lactofen conversion can differentiate between the enantiomers. Previous studies have shown that the herbicidally active *S*-(+)-lactofen was preferentially degraded either in soil or sediment, resulting in relative enrichment of the *R*-(−)-form [15, 16]. However, studies on enantioselectivity of biotransformation enzyme involved in lactofen metabolism by microorganisms are limited. In this study, we demonstrated the occurrence of enantioselectivity in biotransformation of lactofen by LacH and the rising of ER indicated the *R*-(−)-lactofen was preferentially degraded over the *S*-(+)-form.

## Conclusion

Up to now, little is known about the functional enzyme involved in the degradation of lactofen by pure culture. In this paper, a novel esterase LacH was functionally expressed and characterized from *Brevundimonas* sp. LY-2. This is the first report of a functional enzyme involved in the degradation of diphenylether herbicide. In this paper, the enantioselectivity of LacH during lactofen hydrolysis was studied, and the results show that *R*-(−)-lactofen was degraded faster than *S*-(+)-lactofen, indicating the occurrence of enantioselectivity in the enzymatic reaction, which may help in understanding the mechanism of enantioselective degradation of chiral pesticides. In the future, for a better understanding of enantioselective degradation, detailed studies on recognition mechanism of enzymes to differentiate between enantiomers will be needed.

## Methods

### Chemicals

Lactofen (99% purity) was purchased from Sigma-Aldrich Chemical Co. (Shanghai, China). All the *p*-nitrophenyl esters were purchased from Sigma. Methanol, *n*-hexane, and isopropanol were of pure chromatographic grade. All other chemicals used were of analytical grade.

### Bacterial strains and plasmids

*Brevundimonas* sp. LY-2 was deposited in our laboratory. *E. coli* DH5α and *E. coli* BL21 (DE3) were used as the host for gene cloning and protein expression, respectively. The pUC118 and pET-29a (+) were used to construct the genomic library and express the target protein, respectively.

### Screening of genomic library

DNA manipulation was performed as described by Sambrook and Russell [27]. To construct the genomic library, genomic DNA of *Brevundimonas* sp. LY-2 was subjected to partial digestion with *Sau*3AI. Fractions containing approximately 2–4 kb DNA fragments were pooled, ligated into the *Bam*HI site of the plasmid pUC118, and transformed into *E. coli* DH5α. The library was plated onto LB agar containing 100 mg L^−1^ ampicillin and 100 mg L^−1^ lactofen, and incubated at 37 °C for approximately 24 h. The transformants harboring the plasmid containing the functional gene and producing transparent halos were screened and further tested by HPLC analysis to determine their lactofen-degrading ability. Positive recombinant plasmids were extracted and submitted for sequencing.

### Sequence analysis

Nucleotide and deduced amino acid sequence analyses were performed using Omiga Software 2.0. BlastN and BlastP were used for the nucleotide sequence and deduced amino acid identity searches (http://www.ncbi.nlm.nih.gov/Blast), respectively. The signal peptide of the amino acid sequence was predicted using SignalP 4.1 (http://www.cbs.dtu.dk/services/SignalP/). Multiple sequence alignment was performed with Clustal W program [28]. Phylogenetic analysis was carried out via the neighbor-joining method using MEGA 6.0. Bootstrapping of 1000 replicates was performed to estimate the confidence levels of phylogenetic reconstructions [29].

### Gene expression and purification of the recombinant LacH

The open reading frame (ORF) of *lacH* without the signal peptide was amplified by PCR using primers F1 (5′-GGAATTCCATATGGCGACCGCCGAACCGG-3′; *Nde*I site was underlined) and R1 (5′-CCGCTCGAGGTCCGCCAGGAACCGGTC-3′; *Xho*I site was underlined). The PCR product, digested by *Nde*I and *Xho*I, was inserted into pET-29a (+) and the recombinant plasmid was then transformed into *E. coli* BL21 (DE3). The fusion protein was obtained when cells in mid-log phase (optical density at 600 nm = 0.6) were induced with 1.0 mM isopropyl-β-d-thiogalactopyranoside at 30 °C for 3 h. The harvested cells were washed and disrupted by sonication. Cell debris was removed by centrifugation. The supernatant was loaded onto a His-Bind resin (Novagen). The target protein was eluted with 100 mM imidazole after elution of non-target proteins with 25 and 50 mM imidazole. The enzyme was dialysis against 50 mM sodium phosphate buffer (pH 7.0) for 24 h, and concentrated using an Amicon ultrafiltration tube. The protein concentration was quantified by the Bradford method using bovine serum albumin (BSA) as the standard. The purified protein was analyzed by 12% SDS-PAGE gel electrophoresis. The molecular mass markers used were rabbit muscle phosphorylase *b* (97.2 kDa), BSA (66.4 kDa), hen egg white ovalbumin (44.3 kDa), bovine carbonic anhydrase (29.0 kDa), soybean trypsin inhibitor (20.1 kDa), and hen egg white lysozyme (14.3 kDa). The molecular mass of the native protein was determined by gel filtration on a Superdex 200 column. The molecular mass markers used were thyroglobulin (669 kDa), ferritin (440 kDa), bovine gamma globulin (158 kDa), chicken ovalbumin (44 kDa), carbonic anhydrase (29 kDa), and ribonuclease A (13.7 kDa).

### Biochemical characterization

The optimal temperature for lactofen degradation by pure enzyme was determined in 50 mM sodium phosphate buffer (pH 7.0) at different temperatures from 15 to 70 °C. To determine thermostability, the enzyme was pre-incubated at various temperatures ranging from 15 to 80 °C for 30 min, and the residual activity was determined. The optimal pH was measured at 40 °C with pH ranging from 4.0 to 10.0. The buffers used were 50 mM citric acid–sodium citrate (pH 4.0–pH 6.0), sodium phosphate (pH 6.0–pH 8.0), Tris–HCl (pH 8.0–pH 9.0), and glycine–NaOH (pH 9.0–pH 10.0). To investigate the effects of metal ions on enzyme activity, the enzyme was pre-incubated with various metal salts at 40 °C for 30 min. Metal salts (FeSO_4_, CdCl_2_, CaCl_2_, MnCl_2_, MgSO_4_, CuSO_4_, ZnSO_4_, HgCl_2_ and NiSO_4_) were added at a final concentration of 1 mM. The residual activity was assayed and expressed as a percentage of the activity obtained in the absence of added metal ions.

### Substrate specificity and kinetics study

Substrate specificity of LacH was determined using lactofen, *p*-nitrophenyl acetate (C_2_), *p*-nitrophenyl butyrate (C_4_), *p*-nitrophenyl caproate (C_6_), *p*-nitrophenyl caprylate (C_8_), and *p*-nitrophenyl laurate (C_12_) as substrates. Hydrolytic activity toward *p*-nitrophenyl esters was assayed in accordance with the method described by Gao et al. [30]. Hydrolytic activity toward lactofen was performed as described below. For kinetic studies, stock solutions of each substrate were appropriately diluted into five different concentrations around the *K*_m_ values. Kinetic values were obtained from Lineweaver–Burk plots against various substrate concentrations.

### HPLC–MS/MS analysis of lactofen degradation by LacH

Hydrolysis of lactofen was assayed in 50 mM sodium phosphate buffer (pH 7.0) at 40 °C with a final concentration of 50 mg L^−1^ lactofen. Briefly, 1.5 μL of lactofen stock solution (100,000 mg L^−1^, dissolved in methanol) was added to 3 mL sodium phosphate buffer (50 mM, pH 7.0). The reaction was initiated by the addition of 50 μL of the purified LacH (57.5 μg mL^−1^) and incubation for 15 min. The substrate residues were measured by HPLC. Control samples containing boiled enzyme solution were treated and analyzed in the same way. The metabolite of lactofen degradation by LacH was further confirmed by MS/MS [17].

### Chemical analysis

For lactofen determination, the solution mixture was extracted with an equal volume of dichloromethane. The organic layer was dried over anhydrous Na_2_SO_4_, and dichloromethane was removed using a stream of nitrogen at room temperature. The residues were re-dissolved in methanol. All samples were analyzed by HPLC equipped with a Zorbax C-18 ODS Spherex column (250 mm × 4.6 mm). The mobile phase was pure methanol and the flow rate was 1 mL min^−1^. The UV wavelength for detection was 230 nm, and the injection volume was 20 μL.

The lactofen enantiomers were separated and quantified using HPLC with a Chiralcel OD-H column (250 mm × 4.6 mm). The mobile phase was *n*-hexane–isopropanol (97:3, vol/vol), and the flow rate was 1 mL min^−1^. The UV wavelength for detection was 230 nm, and the injection volume was 20 μL.

### Nucleotide sequence accession number

The nucleotide sequence of the *lacH* gene was deposited in the GenBank database under accession number KF286657.

### Strain deposition

*Brevundimonas* sp. LY-2 was deposited to the China General Microbiological Culture Collection Center (CGMCC3651).

### Statistical analysis and reproducibility

All experiments were performed in triplicate. The values were expressed as mean ± SD in the figures.

## Additional file

**Additional file 1: Figure S1.** HPLC–MS/MS analysis of lactofen hydrolyzed by LacH. HPLC spectrum of the hydrolysis reaction by inactive enzyme (A) and active enzyme (B), respectively. MS/MS spectrum of the metabolite with retention time of 2.615 (C). **Figure S2.** Chiral HPLC analysis of lactofen with UV detection. **Table S1.** The hydrolysis rate of pesticides contained carboxylic acid esters by LacH. All of the assays were measured at pH 7.0 and 40 °C for 15 min. **Table S2.** ER changes during the degradation process of lactofen by *Brevundimonas* sp. LY-2. A, at the initial point of the degradation process, no substrate was degraded; B, C and D, approximately 30, 50 and 95% of the substrate were hydrolyzed, respectively.
