# Supplementary material for: Biochemical characterization of an enantioselective esterase from Brevundimonas sp. LY-2
Source: Microb Cell Fact. 2017 Jun 19;16:112. doi: 10.1186/s12934-017-0727-4 (PMC5477170; doi:10.1186/s12934-017-0727-4)
Supplement: Supplementary file 1 — Additional file 1: Figure S1. HPLC–MS/MS analysis of lactofen hydrolyzed by LacH. HPLC spectrum of the hydrolysis reaction by inactive enzyme (A) and active enzyme (B), respectively. MS/MS spectrum of the metabolite with retention time of 2.615 (C). Figure S2. Chiral HPLC analysis of lactofen with UV detection. Table S1. The hydrolysis rate of pesticides contained carboxylic acid esters by LacH. All of the assays were measured at pH 7.0 and 40 °C for 15 min. Table S2. ER changes during the degradation process of lactofen by Brevundimonas sp. LY-2. A, at the initial point of the degradation process, no substrate was degraded; B, C and D, approximately 30, 50 and 95% of the substrate were hydrolyzed, respectively. [file 12934_2017_727_MOESM1_ESM.doc]

**Additional file 1**

**0.0**

**0.5**

**1.0**

**1.5**

**2.0**

**2.5**

**3.0**

**3.5**

**4.0**

**4.5**

**5.0**

**0**

**25**

**50**

**75**

**100**

**125**

**150**

**175**

**200**

**225**

**250**

**275**

**300**

**mV**

**min**

**3.577**

**A**

lactofen

**mV**

**0.0**

**0.5**

**1.0**

**1.5**

**2.0**

**2.5**

**3.0**

**3.5**

**4.0**

**4.5**

**5.0**

**0**

**25**

**50**

**75**

**100**

**125**

**150**

**175**

**200**

**225**

**250**

**275**

**300**

**min**

**3.589**

**2.615**

**B**

lactofen

**C**


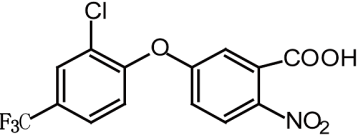


acifluorfen

**Figure S1 HPLC-MS/MS analysis of lactofen hydrolyzed by LacH.** HPLC spectrum of the hydrolysis reaction by inactive enzyme (A) and active enzyme (B), respectively. MS/MS spectrum of the metabolite with retention time of 2.615 (C).


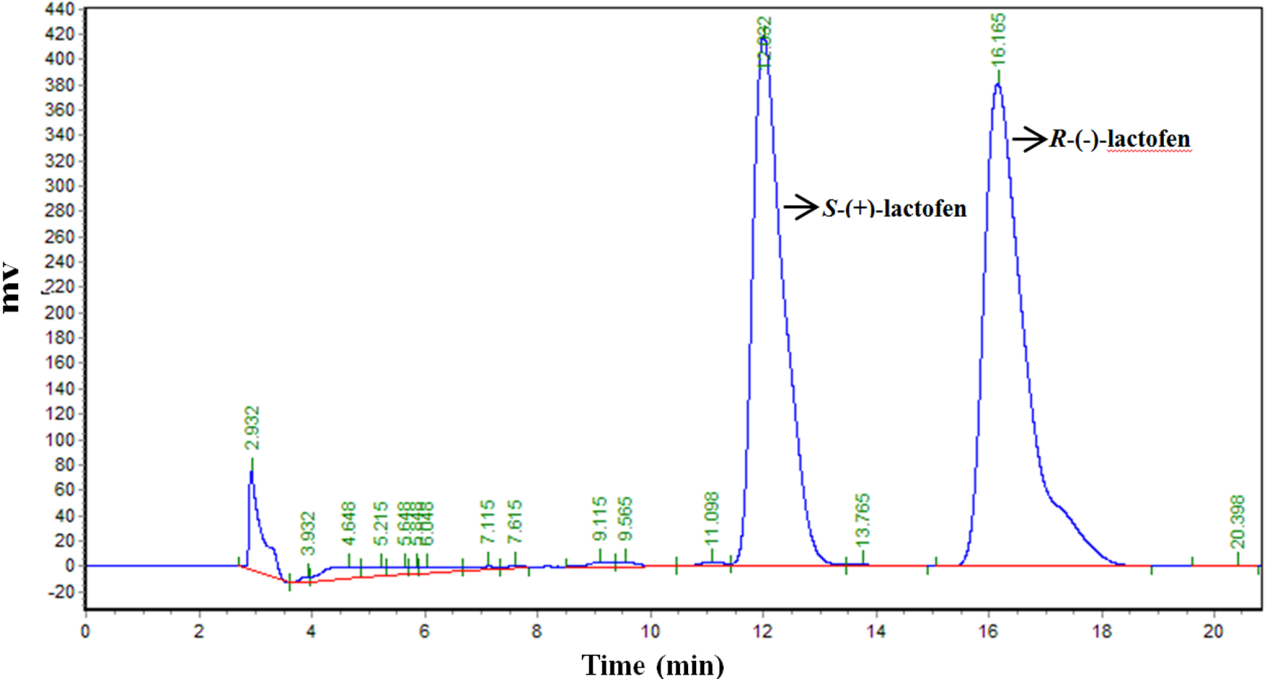


**Figure S2 Chiral HPLC analysis of lactofen with UV detection.**

**Table S1 The hydrolysis rate of pesticides contained carboxylic acid esters by LacH.**

All of the assays were measured at pH 7.0 and 40°C for 15 min.

| Substrate | Hydrolysis rate (%) |
| --- | --- |
| Lactofen | 59.3 |
| Fluazifop-P-butyl | 56.2 |
| Fenoxaprop-P-ethyl | 50.1 |
| Quizalofop-P- ethyl | 23.8 |
| Cyhalofop-butyl | 18.7 |
| Fluoroglycofen | 16.4 |

**Table S2 ER (*S*-(+)-lactofen / *R*-(-)-lactofen) changes during the degradation process of lactofen by *Brevundimonas* sp. LY-2.**

| Sample | A | B | C | D |
| --- | --- | --- | --- | --- |
| ER | 0.90±0.03 | 1.08±0.02 | 1.36±0.02 | 1.61±0.05 |

A, at the initial point of the degradation process, no substrate was degraded; B, C and D, approximately 30% , 50% and 95% of the substrate were hydrolyzed, respectively.
